# Water and Beverage Consumption: Analysis of the Australian 2011–2012 National Nutrition and Physical Activity Survey

**DOI:** 10.3390/nu8110678

**Published:** 2016-10-26

**Authors:** Zhixian Sui, Miaobing Zheng, Man Zhang, Anna Rangan

**Affiliations:** Charles Perkins Centre, School of Life and Environmental Sciences, The University of Sydney, Camperdown 2006, NSW, Australia; miaobing.zheng@sydney.edu.au (M.Z.); mzha7338@uni.sydney.edu.au (M.Z.); anna.rangan@sydney.edu.au (A.R.)

**Keywords:** water intake, dietary pattern, drinking water, diet quality, adults, children

## Abstract

Background: Water consumption as a vital component of the human diet is under-researched in dietary surveys and nutrition studies. Aim: To assess total water and fluid intakes and examine demographic, anthropometric, and dietary factors associated with water consumption in the Australian population. Methods: Dietary intake data from the 2011 to 2012 National Nutrition and Physical Activity Survey were used. Usual water, fluid and food and nutrient intakes were estimated from two days of dietary recalls. Total water includes plain drinking water and moisture from all food and beverage sources; total fluids include plain drinking water and other beverages, but not food moisture. Results: The mean (SD) daily total water intakes for children and adolescents aged 2–18 years were 1.7 (0.6) L for males and 1.5 (0.4) L for females, and for adults aged 19 years and over were 2.6 (0.9) L for males and 2.3 (0.7) L for females. The majority of the population failed to meet the Adequate Intake (AI) values for total water intake (82%) and total fluids intake (78%) with the elderly at highest risk (90%–95%). The contributions of plain drinking water, other beverages and food moisture to total water intake were 44%, 27%, and 29%, respectively, among children and adolescents, and 37%, 37% and 25% among adults. The main sources of other beverages were full-fat plain milk and regular soft drinks for children and adolescents, and tea, coffee, and alcoholic drinks for adults. For adults, higher total water intake was associated with lower percent energy from fat, saturated fat, and free sugars, lower sodium and energy-dense nutrient poor food intakes but higher dietary fibre, fruit, vegetable, caffeine, and alcohol intakes. No associations were found between total water consumption and body mass index (BMI) for adults and BMI *z*-score for children and adolescents. Conclusion: Reported water consumption was below recommendations. Higher water intakes were suggestive of better diet quality.

## 1. Introduction

Water is an essential nutrient required for most of the body’s functions. Evidence suggests that severe dehydration is associated with various clinical conditions, including impaired mental and physical performance, hypertension, urolithiasis, stroke, and certain cancers [1,2]. Even mild dehydration has been shown to impair cognitive functioning, alertness, and exercise capacity [3,4,5]. Despite the consistent evidence linking low water consumption and adverse health outcomes, water intake estimation in free-living populations with ad libitum access to water is lacking.

Recommended intakes for water have been set by a number of organizations although there is no single level of water intake that would ensure adequate hydration and optimal health in all environmental conditions. Requirements vary widely according to environmental conditions, physical activity, dietary intake and individual metabolism. The World Health Organization (WHO) guidelines [6] recommend 2.9 L per day for males and 2.2 L for females to maintain hydration, assuming average sedentary adults under average conditions. In reality, there is substantial scientific evidence relating to differences in water intake across gender, age, anthropometric, and socio-economic subgroups [7,8,9,10].

In Australia, the recommended Adequate Intake (AI) for total water (both fluids and food moisture) and total fluids (excluding food moisture) are set by the National Health and Medical Research Council (NHMRC) and are derived from the median intake estimates from the National Nutrition Survey [2]. The AIs for total water intake (including food moisture) for adults is 3.4 L for males, 2.8 L for females and 1.4–2.2 L for children/adolescents depending on age and gender [2]. The AIs for total fluids are set at 2.6 L for adult males, 2.1 L for adult females, and 1.0–1.9 L for children/adolescents.

The Australian Dietary Guidelines (ADG) encourage water as the fluid of choice and as a general guide for fluids, suggest about 4–5 cups of fluids a day for children up to 8 years, about 6–8 cups for adolescents, 8 cups for women (9 cups in pregnancy and lactation) and 10 cups for men [11]. There is currently little available data on water and fluid intake, beverage sources of intake, and whether recommendations are being met in the Australian population. Although many commonly consumed fluids provide water, they may also be acidic such as low-joule soft drink or contain added sugar, alcohol or caffeine. Recent evidence from the U.S. suggests that plain drinking water consumption is associated with better diet quality [12], and substituting water for sugary beverages is associated with reduced energy consumption and improved body weight management in both children/adolescents and adults [13,14]. However, knowledge of the role of water consumption on diet quality in the Australian population remains limited.

The present study aimed to assess total water and fluid intakes in a nationally representative sample of the Australian population and to examine demographic, anthropometric, and dietary correlates of total water consumption. The secondary aim was to examine the relationship between plain drinking water consumption and dietary factors. These findings will provide fundamental information for future guidelines and may assist further research in the area of water consumption and diet quality, demographic features, and specific health outcomes.

## 2. Materials and Methods

### 2.1. Respondents and Water Intake Data Collection

The present study analysed data from the National Nutrition and Physical Activity Survey (NNPAS) 2011–2012, undertaken by the Australian Bureau of Statistics (ABS). The survey was conducted between May 2011 and June 2012, in adults and children/adolescents aged two years and over. Ethics approval for the survey was granted by the Australian Government Department of Health and Ageing Departmental Ethnics Committee in 2011. Further details about the scope and the methodology of the survey are available from the NNPAS Users Guide [15]. A total of 12,153 respondents were interviewed face-to-face for the collection of dietary intake data using an Automated Multiple-Pass 24-h recall [15] and a second 24-h recall was collected from 7735 respondents via a telephone interview. Respondents were specifically probed regarding water intake and other beverages. After each 24-h recall the respondents were asked additional questions about the intake and main source of plain drinking water. This systematic process has been validated to be effective in maximizing the respondents’ ability to recall and report foods eaten in the previous 24 h [15]. For children under 15 years of age, parents/guardians were used as proxies. Where permission was granted by a parent/guardian, adolescents aged 15–17 years old were interviewed in person. If permission was not granted, questions were answered by an adult [15]. The validity of proxy report for children’s 24-h dietary intake data collection has been reported previously [16]. A food composition database, AUSNUT 2011–2013, developed specifically for NNPAS 2011–2012, was used to estimate water, fluid, food moisture, foods, beverages, and nutrient intakes [17].

### 2.2. Water, Moisture, and Food Sources

In this study, intake of total water was defined as plain drinking water plus other beverages, and food moisture, in line with the descriptions in the Nutrition Reference Values for Australia and New Zealand [2]. Plain drinking water included tap water (domestic tap, tank or rain water) and bottled water (packaged with- and without fortified water). Other beverages included moisture obtained from tea (regular, decaffeinated and herbal tea), coffee (regular, decaffeinated and coffee beverage), fruit/vegetable juice (freshly and commercially prepared, fortified fruit juice), fruit drinks (ready to drink, prepared from concentrated and dry powder), regular/diet cordial, regular/diet soft drinks, energy/sport drinks, full/reduced fat milk, flavoured milk, milk substitute, alcoholic drinks, and other flavoured and non-flavoured beverage drinks. Water used to dilute concentrated drinks was categorized according to the beverage type. Milk added to tea, coffee, and breakfast cereal was separated and categorized according to milk type.

### 2.3. Anthropometry, Demographic, and Other Characters

Respondents’ weight, height, and waist circumference (WC) were objectively measured. Body Mass Index (BMI) for adults was calculated as weight (kg) divided by height squared (m^2^). BMI *z*-scores for children/adolescents were calculated using World Health Organization age-and gender-specific growth charts [18]. Age groups were categorized based on the NNPAS age categories (2–3, 4–8, 9–13, 14–18,19–30, 31–50, 51–70, and 70+ years) and socio-economic quintiles were based on the Socio-Economic Index of Disadvantage for Areas (SEIFA), where the first SEIFA quintile indicates the most disadvantaged areas [19]. Lifestyle factors including total minutes of physical activity during the past week and total sleep duration the day prior to the interview were self-reported at the time of interview.

### 2.4. Dietary Factors

The term “core food groups” as used in this study refers to grains, vegetables, fruits, dairy products, and meat and alternatives, as described in the Australian Guide to Healthy Eating (AGHE) [11,20]. To assess consumption of all foods within a food group, all individually recorded food items and foods as part of a mixed dish were included. The individual food components from a mixed dish were estimated using the AUSNUT 2011–2013 recipe file [17] and were classified under their respective core food group. Discretionary foods (solid) such as cakes, biscuits, confectionary, deep-fried fast foods, and processed meat, and discretionary beverages such as soft drinks, fruit drinks and alcoholic drinks are defined as “foods high in saturated fat and/or added sugars, added salt or alcohol and low in fibre” and were categorized accordingly [19].

### 2.5. Misreporting

Misreporting has been identified in the NNPAS 2011–2012 survey, with 16%–26% of the respondents classified as under-reporting total energy intake [19]. To enable a more accurate interpretation of dietary data, it has been suggested that analysis be conducted with and without potential under- and over-reporters [21,22,23]. Plausible reporters were identified based on the Goldberg cut-off (energy intake: basal metabolic rate 0.92–2.17 for usual intake from 2 days’ data) [24]. The Goldberg cut-offs have been validated for use with data from 24-h recalls [25]. In this paper, only the results from plausible-reporters are presented.

### 2.6. Statistical Analysis

**Figure 1:**
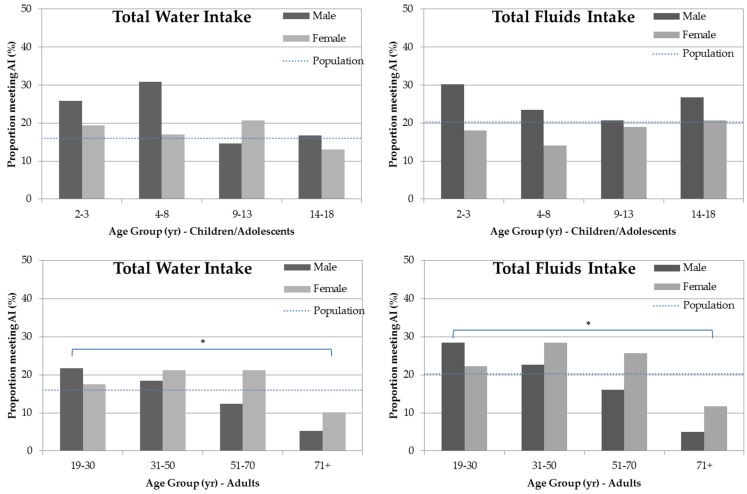
The proportion of the respondents (%) meeting the adequate intake (AI) for total water intake and total fluids intake. * significant trend with age (ANOVA) (*p* < 0.001).

**Figure 2:**
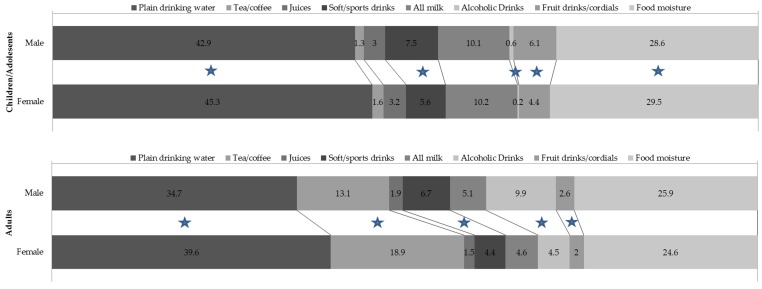
Proportion of contribution by sources of total water intake (%) by gender (Star indicates a significant difference (*p* < 0.05) between genders).

Statistical analyses were performed using SPSS for Windows 22.0 software (IBM Corp. Released 2013. IBM SPSS Statistics for Windows, Version 22.0., Armonk, NY, USA). Respondent’s usual intakes of plain drinking water, other beverages, food moisture, core food groups, and discretionary foods/beverages from the two 24-h recalls were analysed using the Multiple Source Method [26] (presented in Table 1, Table 2 and Table 4 and Figure 1). Only the first day of recall was used to examine proportions of contributions to total water intake (Table 3 and Figure 2). Descriptive statistics were used to report the total water intake according to different characteristics. Children/adolescents and adults were analysed separately and also by gender due to the different recommended intakes [2]. Data were presented as the per capita mean and standard deviation (SD), median and the 25th and 75th percentiles, or as a percentage (%). Total water intake was categorized into quartiles to assess respondents’ characteristics and dietary intakes. Chi-square tests and ANOVA analysis were used to assess the differences between proportions and to compare mean differences in consumption and linear trends where appropriate. Median differences were compared using Kruskal-Wallis tests. Multiple linear regression methods were used to examine the relationship between usual plain drinking water intake and covariates including gender, age, socio-economic status, and dietary factors, adjusted for total energy intake and physical activity level. For all tests, a *p*-value of < 0.05 was considered statically significant.

## 3. Results

### 3.1. Total Water Intake

Data presented in Table 1 show total water intake in children/adolescents and adults, by gender and age groups. Per capita total water intakes for children/adolescents were 1.7 L and 1.5 L for boys and girls, respectively, and for adults were 2.6 L and 2.3 L for males and females, respectively. Total water intake increased with older age in children/adolescents (ANOVA trend *p* < 0.001) but declined for adults (*p* < 0.001). Large variation was observed in the inter-quartile ranges for fluids intake particularly for adolescent boys and adult males.

Total water intake and total fluids intake were compared to age and gender specific AI values (Figure 1). Overall, about 18% of the respondents met AI for total water, and 22% met AI for total fluids. These proportions did not vary significantly between age groups for children/adolescents, but declined with increasing age in adults (*p* < 0.001) with only 5% of men and 10% of women aged over 71 years meeting the AI.

Table 2 presents the demographic, anthropometric, lifestyle, and dietary characteristics by quartile of total water intake. Age, gender, season of interview, country of birth and socioeconomic status were significantly associated with total water intake among children/adolescents and/or adults, and were thus adjusted for in subsequent analysis.

Among children/adolescents, total water intake was positively associated with waist circumference, but not BMI *z*-score. Children/adolescents reporting higher total water intake had greater intakes of energy, protein (as percent energy, %E), dietary fibre (%E), and fruit (g/Mj) and dairy products (g/Mj) but lower total fat (%E), saturated fat (%E), free sugar (%E), sodium (mg/Mj), and discretionary foods (g/Mj).

Among adults, being born in non-English speaking countries and categorized in the lowest socio-economic quintile were associated with lower total water intake. No BMI gradient for total water intake was observed. Adults reporting higher total water intake also reported higher intakes of energy, dietary fibre (%E) and caffeine (mg/Mj) but lower intakes of total fat (%E), saturated fat (%E), total carbohydrates (%E), free sugar (%E), sodium (mg/Mj). Higher food densities of vegetable, fruits, dairy products, and alcoholic drinks but lower densities of grains and discretionary foods/beverages were also reported by those with higher total water intakes.

### 3.2. Sources of Water

The contributions from different sources to total water intake are presented in Table 3. Plain drinking water, mostly obtained from tap water, was the most common source of total water intake, but children/adolescents and adults reported different choices of other beverages.

Among children/adolescents the contributions of plain drinking water, other beverages, and food moisture to total water intake were 44.1%, 26.9%, and 29.0%, with full-fat plain milk being the most common other beverage followed by regular soft drinks and fruit juice. Analysis of different sources of total water intake by age indicated that older children/adolescents reported a higher proportion of total water from tea/coffee, soft/sports drinks, and alcoholic drinks but a lower proportion from juice, fruit drinks/cordials and milk.

Among adults, the proportions of plain drinking water, other beverages, and food moisture to total water intake were 37.4%, 25.2%, and 37.4%. Apart from plain drinking water, tea, coffee, and alcoholic drinks were the largest contributors to total water intake, with older adults reporting larger proportions of total water intake from tea, coffee, reduced fat and skim milk, and food moisture compared with younger adults.

Figure 2 summarises the principal sources of total water intake by gender. Among children/adolescents, girls reported higher proportions from plain drinking water and food moisture compared to boys. Boys reported larger proportions of soft/sports drinks, fruit drinks/cordials, and alcoholic drinks than girls. Among adults, females were more likely than males to consume plain drinking water and tea/coffee, whereas males consumed higher proportions of soft/sports drinks, fruit drinks/cordials, and alcoholic drinks than females. Notably, adult males reported higher intakes of other beverages (39.4% total water intake) than plain drinking water (34.7%) (*p* < 0.001).

### 3.3. Plain Drinking Water

Among children/adolescents, plain drinking water intake was associated with being female, older age, and lower consumption of dairy products and discretionary beverages/foods (Table 4). Among adults, plain drinking water intake was higher in females and those of higher socio-economic status, but decreased with age. Plain drinking water was also associated with higher consumption of fruit, but lower consumption of grains, dairy products and discretionary foods/beverages. Analyses were adjusted for energy intake, physical activity, country of birth, season of interview, BMI *z*-score for children/adolescents, and BMI for adults (Table 4).

## 4. Discussion

These analyses based on a representative sample of Australian population showed estimated total water intakes to be 1.5–1.7 L for children/adolescents, and 2.3–2.6 L for adults. Plain drinking water was the most commonly consumed beverage type for Australian children/adolescents and adults, in line with Dietary Guideline recommendations. Full-fat plain milk and regular soft drinks were the main contributors to other beverages for children/adolescents, and tea, coffee, and alcoholic drinks for adults. Total water intake was higher in males than females, and in older age groups in children/adolescents and younger age groups in adults. Plain water intake was higher in females than males, and was inversely associated with the consumption of dairy products and discretionary foods/beverages in adults.

Our data can be compared to similar national nutrition surveys from the US and European countries. In the US National Health and Nutrition Examination Survey (NHANES) 2005–2012, per capita total water intake for children/adolescents was 1.6–1.7 L, similar to our findings, and 2.2–3.5 L for adults with large gender and age variations [7,8]. Total water intake in French children aged 4–13 years was 1.3 L [27], and adults reported 2.1–2.5 L in the Irish national survey in 2008–2010 [10]. These surveys used 24-h recalls or food records to estimate water intake.

The amounts of total water intake and total fluids intake were compared to AI values by gender and age group and showed that the majority of the respondents failed to meet the recommended total water intake (82%) and total fluids intake (78%). The Australian Nutrition Reference Values acknowledge that there is no single level of water intake that would ensure adequate hydration and optimal health for the whole population. Thus, the total water and total fluids AI values were based on the median intake from the National Nutrition Survey 1995 and were set at the level of the highest median intake from any of the four age categories for each gender [28] and therefore represent only crude guidelines.

The sub-group that was least likely to meet the AI values were older adults aged over 70 years (5% for males and 10% for females). The average shortfall was 1.2 L for males and 0.8 L for females. Older adults are at risk of dehydration due to decreased perception of thirst, inadequate fluid intake, a decline in kidney function and increased use of diuretics and laxatives in this age group. The outcomes of dehydration in the elderly are serious and include cognitive impairment, functional decline, falls or stroke [11], and close monitoring of water consumption in this age group is warranted [29].

We found that season of interview, physical activity level, socio-economic status, and gender were associated with total water intake, in agreement with other research [7,8,10]. Our findings also showed that BMI in adults or BMI *z*-score in children/adolescents was not associated with total water intake or plain drinking water intake. Similar observations were reported in the US, where BMI was associated with beverage intake but not plain drinking water in adults aged 20 years and over [9].

Higher total water consumption was associated with better diet quality, indicated by higher intakes of dietary fibre, fruit and vegetables, and lower intakes of fat, saturated fat, free sugars, sodium and discretionary foods. In addition, higher consumption of plain drinking water, which comprised more than one-third of total water intake, was also associated with lower consumption of discretionary choices, confirming that promoting water intake could be a useful public health strategy. As the majority of plain drinking water was consumed as tap water (95% in children/adolescents, 90% in adults), which is fluoridated in Australia, additional benefits are conferred for the development of strong teeth and bones.

Food moisture is an important source of total water intake in the population, accounting for about 30% of total water intake in children/adolescents and 25% in adults. The water content of food is one of the major determinants of dietary energy density. Dietary energy density has been positively associated with obesity, diabetes and inversely associated with dietary quality [30,31]. Although Australia has not developed a specific recommendation for the optimal dietary water-to-energy ratio, this substantial contribution of food moisture suggests that assessing fluid intake without considering food intake would provide misleading results.

Our results highlight the high consumption of sugar-sweetened beverages consumption by children/adolescents, especially adolescents, where soft/sports drinks contribute to over 10% of total water intakes (approximately 14% of total fluids intake). Our analysis shows that these beverages replace milk and juice in the older age groups. Sugar-sweetened beverages have been shown to have a detrimental impact on health [32,33,34]. The effects of increased consumption of certain beverages on health outcomes have been well documented. Past studies have focused on the contribution of beverages to energy and nutrient intakes [9,35], and examined the potential beneficial outcomes of replacing sugar-sweetened beverages with plain water or more nutrient-dense options such as milk [13,32,36]. Our results show that there is considerable room for further improvement in fluid intakes of Australian children/adolescents. Among adults, the major sources of other beverages were tea and coffee, followed by alcohol. Tea and coffee are suitable alternatives to plain drinking water but the caffeine content may have unwanted stimulant effects in susceptible people [11,37,38].

The present analysis had several limitations. First, our results are derived from cross-sectional data and causal relationships cannot be inferred. Second, recall bias is a serious limitation in the collection of dietary intake data and under-reporting or selective reporting of discretionary foods and beverages is common. The recall of water intake is particularly challenging using self-report methods but a number of additional questions were asked during the interview to encourage best possible estimates. Third, biomarkers of hydration status were not available to assess hydration status in this population as these methods tend to be expensive and time-consuming [39]. However, ongoing research suggests that urinary biomarkers such as 24-h urine volume and osmolality are strongly correlated with total fluid intake in normal daily living conditions and may be useful in future studies of fluid intake [40]. Lastly, proxy recall for younger children may be an additional source of error. Despite these limitations, these data have a number of advantages as they represent a large, nationally representative data source that forms the basis for dietary surveillance in Australia. In addition, we used “usual intake” data based on two days of recall rather than one day only, which may result in slightly lower estimated intakes [15,41].

## 5. Conclusions

This study provides valuable new data on the consumption of water and other beverages in a sample representative of the Australian population. Reported water consumption was below recommendations, particularly for the elderly population who may be at higher risk of inadequate hydration. Additionally, our results showed that higher intakes of plain drinking water were associated with positive dietary features, thus making water an optimal beverage choice. Given the interest in understanding the association of water intake with a variety of health outcomes, these findings provide fundamental information to develop effective health promotion policies and campaigns for the Australian population.

## Figures and Tables

**Table 1:** Total water and total fluids intakes (L) by gender and age groups.

**Children/Adolescents**		**Age (Years)**	**2–18**	**2–3**	**4–8**	**9–13**	**14–18**	*p*-Value *
Males		*n*	1017	159	295	294	269	
	Total water	AI		1.4	1.6	2.2	2.7	
		Mean (SD)	1.7 (0.6)	1.2 (0.4)	1.5 (0.4)	1.7 (0.5)	2.1 (0.6)	<0.001
		Median (IQR)	1.6 (1.3–2.0)	1.2 (1.0–1.4)	1.4 (1.2–1.7)	1.7 (1.4–2.0)	2.0 (1.6–2.5)	<0.001
	Total fluids	AI		1.0	1.2	1.6	1.9	
		Mean (SD)	1.2 (0.5)	0.9 (0.3)	1.0 (0.4)	1.3 (0.4)	1.6 (0.6)	<0.001
		Median (IQR)	1.1 (0.9–1.5)	0.8 (0.6–1.1)	1.0 (0.8–1.2)	1.2 (1–1.5)	1.5 (1.1–1.9)	<0.001
	Food moisture	Mean (SD)	0.4 (0.2)	0.4 (0.1)	0.4 (0.1)	0.5 (0.1)	0.5 (0.2)	<0.001
		Median (IQR)	0.4 (0.3–0.5)	0.3 (0.3–0.4)	0.4 (0.3–0.5)	0.4 (0.4–0.6)	0.5 (0.4–0.6)	<0.001
Females		*n*	953	160	276	295	222	
	Total water	AI		1.4	1.6	1.9	2.2	
		Mean (SD)	1.5 (0.4)	1.1 (0.3)	1.3 (0.3)	1.3 (0.3)	1.7 (0.5)	<0.001
		Median (IQR)	1.4 (1.1–1.7)	1.1 (0.9–1.3)	1.2 (1.1–1.5)	1.6 (1.3–1.8)	1.7 (1.4–2)	<0.001
	Total fluids	AI		1.0	1.2	1.4	1.6	
		Mean (SD)	1.0 (0.4)	0.8 (0.3)	0.9 (0.3)	1.1 (0.4)	1.3 (0.4)	<0.001
		Median (IQR)	1.0 (0.8–1.3)	0.7 (0.6–0.9)	0.8 (0.7–1.1)	1.1 (0.9–1.3)	1.2 (1.0–1.5)	<0.001
	Food moisture	Mean (SD)	0.4 (0.1)	0.4 (0.1)	0.4 (0.1)	0.5 (0.1)	0.5 (0.2)	<0.001
		Median (IQR)	0.4 (0.3–0.5)	0.4 (0.3–0.4)	0.4 (0.3–0.5)	0.4 (0.4–0.5)	0.4 (0.3–0.5)	<0.001
**Adults**		**Age (Years)**	**19+**	**19–30**	**31–50**	**51–70**	**71+**	***p*-Value**
Males		*n*	2999	553	1160	925	361	
	Total water	AI	3.4	3.4	3.4	3.4	3.4	
		Mean (SD)	2.6 (0.9)	2.8 (1.0)	2.7 (0.9)	2.5 (0.8)	2.2 (0.6)	<0.001
		Median (IQR)	2.4 (2.0–3.0)	2.6 (2.1–3.3)	2.5 (2.1–3.1)	2.4 (2–2.9)	2.1 (1.8–2.5)	<0.001
	Total fluids	AI	2.6	2.6	2.6	2.6	2.6	
		Mean (SD)	2.0 (0.8)	2.2 (0.9)	2.1 (0.8)	1.9 (0.7)	1.6 (0.5)	<0.001
		Median (IQR)	1.8 (1.4–2.4)	2.0 (1.6–2.7)	2.0 (1.6–2.5)	1.8 (1.4–2.3)	1.5 (1.2–1.8)	<0.001
	Food moisture	Mean (SD)	0.6 (0.2)	0.6 (0.2)	0.6 (0.2)	0.6 (0.2)	0.6 (0.2)	ns
		Median (IQR)	0.6 (0.4–0.7)	0.5 (0.5–0.7)	0.5 (0.4–0.7)	0.6 (0.5–0.7)	0.6 (0.5–0.7)	ns
Females		*n*	3233	548	1189	1024	472	
	Total water	AI	2.8	2.8	2.8	2.8	2.8	
		Mean (SD)	2.3 (0.7)	2.2 (0.7)	2.4 (0.7)	2.3 (0.7)	2.0 (0.6)	<0.001
		Median (IQR)	2.2 (1.8–2.7)	2.1 (1.8–2.6)	2.3 (1.9–2.7)	2.2 (1.8–2.7)	2.0 (1.6–2.3)	<0.001
	Total fluids	AI	2.1	2.1	2.1	2.1	2.1	
		Mean (SD)	1.8 (0.6)	1.7 (0.6)	1.9 (0.7)	1.8 (0.6)	1.5 (0.5)	<0.001
		Median (IQR)	1.7 (1.3–2.1)	1.6 (1.3–2.1)	1.8 (1.4–2.2)	1.7 (1.3–2.1)	1.4 (1.1–1.8)	<0.001
	Food moisture	Mean (SD)	0.5 (0.2)	0.5 (0.2)	0.5 (0.2)	0.5 (0.2)	0.5 (0.2)	ns
		Median (IQR)	0.5 (0.4–0.6)	0.5 (0.4–0.6)	0.5 (0.4–0.6)	0.5 (0.4–0.6)	0.5 (0.4–0.6)	ns

* *p*-value for trends analysis (ANOVA) for means; Kruskal-Wallis tests for medians; ns: not statistically significant; AI: Adequate Intake (L/day); IQR: 25th–75th percentile; SD: Standard Deviation.

**Table 2:** Anthropometric, demographic, and lifestyle characteristics by quartile of total water intakes in children/adolescents and adults.

Quartiles	Children/Adolescents					Adults				
	Q1 (<1.2)	Q2 (1.2–1.5)	Q3 (1.5–1.8)	Q4 (>1.8)	*p*-Value *	Q1 (<1.9)	Q2 (1.9–2.3)	Q3 (2.3–2.8)	Q4 (>2.8)	*p*-Value *
Mean total water intake (L)	1.0 (0.1)	1.3 (0.1)	1.7 (0.1)	2.3 (0.4)	<0.001	1.6 (0.2)	2.1 (0.1)	2.5 (0.1)	3.5 (0.7)	<0.001
**Demographic**										
Age (year)	5.7 (3.9)	8.4 (4.5)	10.3 (4.4)	12.9 (3.4)	<0.001	52.1 (19.4)	49.8 (17.5)	48.3 (16.6)	44.9 (15.3)	<0.001
Male (%)	39.1	50.2	51.7	65.4	<0.001	37.0	44.0	50.4	61.0	<0.001
Interviewed in summer (%)	25.4	24.6	26.8	32.7	<0.001	25.0	28.7	28.8	29.3	<0.001
Born in NE countries (%) ^+^	5.3	5.5	6.5	6.3	ns	19.8	17.1	16.0	13.2	<0.001
Lowest SES quintile (%)	16.6	17.3	16.6	14.8	ns	21.0	16.6	16.8	16.5	0.01
Highest SES quintile (%)	24.1	25.8	28	26	ns	22.7	24.6	25.2	24.0	ns
**Anthropometric ^**										
BMI (*z*-score for children, kg/m^2^ for adults)	0.5 (1.2)	0.4 (1.2)	0.5 (1.1)	0.7 (1.1)	ns	27.1 (0.4)	27.7 (0.4)	27.3 (0.4)	27.5 (0.4)	ns
Waist circumference (cm)	63.9 (0.8)	64.7 (0.8)	64.8 (0.7)	67.3 (0.8)	<0.001	99.9 (4.2)	100.8 (4.2)	97.9 (4.1)	100.3 (4.2)	ns
**Lifestyle ^**										
Physical activity in last week (min)	104.2 (26.3)	213.1 (20.1)	260.2 (11.5)	252.3 (14.1)	ns	199.7 (18)	231.5 (17.9)	251.3 (17.8)	290.1 (18.2)	<0.001
Sleep duration (min)	581.4 (10.8)	593.2 (8.3)	590.4 (7.5)	583.6 (7.6)	ns	479.1 (4.8)	477.2 (4.8)	473.3 (4.8)	465.9 (4.9)	0.001
Currently smoking (%)	-	-	-	-	-	14.3	14.4	14.1	14.3	ns
**Nutrients ^**										
Water density (L/Mj)	0.15 (0.1)	0.19 (0.1)	0.22 (0.1)	0.27 (0.1)	<0.001	0.20 (0.1)	0.25 (0.04)	0.29 (0.1)	0.36 (0.2)	0.001
Energy (Mj)	7.2 (0.2)	7.8 (0.2)	8.3 (0.2)	9.2 (0.2)	<0.001	8.5 (0.1)	9.2 (0.1)	9.6 (0.1)	10.4 (0.1)	<0.001
Protein (%E)	15.9 (0.4)	15.7 (0.4)	16.6 (0.4)	16.7 (0.4)	0.001	17.6 (0.3)	17.7 (0.3)	17.9 (0.3)	18.0 (0.3)	ns
Total fat (%E)	32.4 (0.7)	31.5 (0.6)	31.1 (0.6)	30.3 (0.7)	0.002	32.7 (0.4)	31.8 (0.4)	31.2 (0.4)	29.9 (0.4)	<0.001
Saturated fat (%E)	14.1 (0.4)	14.1 (0.4)	13.7 (0.4)	13.4 (0.4)	0.03	13.3 (0.2)	12.7 (0.2)	12.2 (0.2)	11.7 (0.2)	<0.001
Total carbohydrates (%E)	49.1 (0.8)	50.2 (0.7)	49.5 (0.7)	49.8 (0.8)	ns	43.8 (0.5)	43.4 (0.5)	42.7 (0.5)	41.4 (0.5)	<0.001
Free sugar (%E)	11.9 (2.3)	11.4 (1.8)	10.4 (1.5)	10.3 (1.3)	<0.001	9.5 (1.0)	9.0 (0.9)	8.5 (0.9)	8.2 (0.7)	<0.001
Dietary fibre (%E)	1.9 (0.1)	2.0 (0.1)	2.1 (0.1)	2.2 (0.1)	<0.001	2.0 (0.1)	2.2 (0.1)	2.2 (0.1)	2.3 (0.1)	<0.001
Sodium (mg/Mj)	298.4 (10.3)	285.6 (9.4)	279.4 (9.4)	292 (10.3)	0.04	286.2 (6)	279.1 (6)	276.7 (6)	268.4 (6.1)	0.001
Caffeine (mg/Mj)	2.2 (0.4)	1.9 (0.4)	2.0 (0.4)	2.3 (0.4)	ns	17.0 (1.0)	19.6 (1)	22.0 (1)	23.1 (1.0)	<0.001
**Food groups ^**										
Grains (g/Mj)	22.8 (0.9)	21.7 (0.8)	22.4 (0.8)	22.6 (0.9)	ns	20.8 (0.5)	20.4 (0.4)	20.4 (0.4)	19.7 (0.5)	0.02
Vegetable (g/Mj)	16.4 (0.8)	15.3 (0.7)	16.2 (0.7)	16.4 (0.8)	ns	20.8 (0.5)	21.2 (0.5)	21.9 (0.5)	21.9 (0.5)	0.005
Fruits (g/Mj)	21.8 (1.4)	24 (1.3)	24 (1.3)	24.9 (1.4)	0.03	17.7 (0.7)	19.2 (0.7)	19.4 (0.7)	20.2 (0.7)	<0.001
Dairy products (g/Mj)	30.4 (2.0)	31 (1.8)	32.9 (1.8)	34.7 (2.0)	0.02	25.3 (0.8)	26.4 (0.8)	26.9 (0.8)	26.7 (0.8)	0.048
Meat and alternatives (g/Mj)	12.4 (0.6)	11.6 (0.5)	12.1 (0.5)	12.3 (0.6)	ns	15.1 (0.3)	14.8 (0.3)	14.9 (0.3)	15 (0.3)	ns
Discretionary foods (g/Mj)	23.2 (0.9)	21.7 (0.8)	19.8 (0.8)	20.4 (0.9)	<0.001	18.4 (0.4)	17.3 (0.4)	16.3 (0.4)	16.1 (0.4)	<0.001
Discretionary beverages (g/Mj)	26.7 (2.2)	26.5 (2)	24.1 (2)	25.8 (2.2)	ns	21.7 (0.9)	20.6 (0.9)	19.4 (0.9)	18.1 (1.0)	<0.001
Alcoholic drinks (g/Mj)	-	-	-	-	-	14.7 (1.7)	18.7 (1.7)	22.3 (1.7)	34.3 (1.7)	<0.001

* Trends analysis (ANOVA) for *p*-values for mean; Chi-square tests for *p*-values for proportion; + Born in non-English speaking countries; ^ Adjusted for age, gender, whether born in non-English speaking countries, and season of interview; ns: not statistically significant; %E: percentage energy.

**Table 3:** Proportion of contribution by sources of total water intake (%) by age group (year).

Children/Adolescents	2–18	2–3	4–8	9–13	14–18	*p*-Value *	Adults	19+	19–30	31–50	51–70	71+	*p*-Value
**Plain drinking water**	44.1	39.1	45.1	44.2	44.4	ns	**Plain drinking water**	37.4	43.9	38.7	32.0	29.5	<0.001
Tap water	42.0	38.1	42.7	42.0	42.3	ns	Tap water	33.7	39.4	34.7	28.9	28.1	<0.001
Bottled water	2.1	1.0	2.4	2.2	2.1	ns	Bottled water	3.8	4.5	4.0	3.1	1.3	<0.001
**Tea/coffee**	1.4	0.2	0.4	0.7	3.3	<0.001	**Tea/coffee**	16.2	8.1	16.0	21.2	24.2	<0.001
Tea	0.9	0.2	0.4	0.6	1.6	<0.001	Tea	8.2	4.3	7.2	11.2	14.7	<0.001
Coffee	0.5	<0.1	<0.1	0.1	1.6	<0.001	Coffee	7.9	3.8	8.8	10.1	9.5	<0.001
**Juices**	3.1	3.6	3.4	2.9	2.9	<0.001	**Juices**	1.7	2.0	1.6	1.5	1.6	<0.001
Fruit Juice	3.1	3.5	3.4	2.9	2.9	<0.001	Fruit Juice	1.6	2.0	1.5	1.4	1.4	<0.001
Vegetable Juice	<0.1	<0.1	<0.1	<0.1	<0.1	ns	Vegetable Juice	0.1	<0.1	<0.1	0.1	0.2	ns
**Milk**	10.1	17.4	10.0	9.9	8.5	<0.001	**Milk**	4.8	5.0	4.5	4.8	6.4	<0.001
Plain Milk (Full Fat)	6.3	14.1	6.7	5.8	4.4	<0.001	Plain Milk (Full Fat)	2.1	2.5	1.9	1.9	2.4	ns
Plain Milk (Reduced Milk)	1.7	2.3	1.8	1.8	1.4	ns	Plain Milk (Reduced Milk)	1.5	1.1	1.4	1.7	2.5	<0.001
Plain Milk (Skim)	0.4	0.4	0.4	0.5	0.2	ns	Plain Milk (Skim)	0.6	0.4	0.5	0.7	1.0	<0.001
Flavoured Milk	1.7	0.5	1.2	1.7	2.5	<0.001	Flavoured Milk	0.7	1.0	0.8	0.5	0.4	<0.001
Milk substitute	<0.1	<0.1	<0.1	<0.1	<0.1	ns	Milk substitute	<0.1	<0.1	<0.1	<0.1	<0.1	<0.001
**Soft/sports drinks**	6.6	0.9	3.1	6.8	10.6	<0.001	**Soft/sports drinks**	5.5	7.8	5.8	4.0	1.9	<0.001
Regular Soft Drinks	5.4	0.9	2.9	5.4	8.4	<0.001	Regular Soft Drinks	3.2	5.1	3.2	2.1	1.2	<0.001
Diet Soft Drinks	0.8	<0.1	0.2	1.0	1.4	<0.001	Diet Soft Drinks	1.8	1.7	2.1	1.9	0.6	<0.001
Energy & Sports Drinks	0.4	<0.1	<0.1	0.3	0.9	<0.001	Energy & Sports Drinks	0.4	1.0	0.5	0.1	<0.1	<0.001
**Fruit drinks/cordials**	5.3	6.7	5.8	5.8	3.9	ns	**Fruit drinks/cordials**	2.3	4.1	2.1	1.6	1.9	<0.001
Fruit Drinks	2.4	3.2	2.7	2.4	2	ns	Fruit Drinks	1.0	1.8	0.8	0.6	1.1	<0.001
Regular Cordial	2.1	2.3	2.4	2.5	1.5	ns	Regular Cordial	0.9	1.8	1.0	0.6	0.6	<0.001
Diet Cordial	0.3	0.8	0.3	0.2	0.1	ns	Diet Cordial	0.2	0.2	0.2	0.2	<0.1	ns
Others	0.5	0.3	0.3	0.8	0.4	ns	Others	0.2	0.3	0.1	0.1	0.1	ns
**Alcoholic Drinks**	0.4	<0.1	<0.1	<0.1	1.2	<0.001	**Alcoholic Drinks**	7.0	6.1	7.1	8.3	5.1	<0.001
**Food moisture**	29.0	32.1	32.1	29.8	25.1	ns	**Food moisture**	25.2	23.8	24.2	26.6	29.4	0.02

* Chi-square tests for proportion; ns: not statistically significant.

**Table 4:** Association of plain drinking water intake with dietary covariates.

Plain Drinking Water Intake (mL)		Children/Adolescents			Adults		
		β	SE	*p*-Value	β	SE	*p*-Value
Gender (Ref. Male)		−63.3	19.1	<0.001	−10.2	16.5	0.01
Age (year)		37.3	3.7	<0.001	−8.9	0.4	<0.001
BMI *		2.6	0.7	ns	3.3	0.9	ns
SEIFA (Ref. 1st quintile)	2nd quintile	−0.7	*30.0*	ns	44.3	22.5	0.04
	3rd quintile	62.7	29.4	ns	49.5	22.5	0.03
	4th quintile	38.9	30.0	ns	92.9	23.4	<0.001
	5th quintile	32.1	28.1	ns	69.2	21.8	0.002
Grain (100 g)		−11.0	3.8	ns	−34.3	9.1	<0.001
Vegetables (100 g)		−10.0	20.0	ns	−11.7	9.4	ns
Fruit (100 g)		−6.1	3.9	ns	27.1	6.3	<0.001
Dairy products (100 g)		−30.8	9.8	0.002	−31.2	4.9	<0.001
Meat and alternatives (100 g)		19.1	5.4	ns	13.6	13.7	ns
Discretionary beverages (100 g)		−74.8	9.0	<0.001	−34.7	4.1	<0.001
Discretionary foods (100 g)		−46.4	14.2	0.001	−64.3	10.5	<0.001
Alcoholic drinks (100 g)					−4.0	2.1	ns

Note: β indicates the change of plain drinking water in mL per unit change of the covariates, adjusted for total energy intake, physical activity, whether born in non-English speaking countries, and season of interview; * BMI *z*-score for children/adolescents, and BMI for adults; SE: Standard Error; ns: not statistically significant.
